# Children and Parents’ Awareness Regarding Potential Hazards Derived from the Use of Chemical Products in Greece

**DOI:** 10.3390/ijerph182412948

**Published:** 2021-12-08

**Authors:** Christina Tsitsimpikou, Nikolaos Georgiadis, Konstantinos Tsarouhas, Panagiotis Kartsidis, Eleni Foufa, Flora Bacopoulou, Athanasios Choursalas, Dimitrios Kouretas, Alexandros K. Nikolaidis, Elisabeth A. Koulaouzidou

**Affiliations:** 1 General Chemical State Laboratory of Greece, 11521 Athens, Greece; chtsitsi@yahoo.com (C.T.); elenifoufa@yahoo.gr (E.F.); 2 European Chemicals Agency, 00121 Helsinki, Finland; nikolaos.georgiadis@echa.europa.eu; 3 Department of Cardiology, University Hospital of Larissa, 41110 Larissa, Greece; ktsarouhas14@yahoo.gr; 4 School of Medicine, Faculty of Health Sciences, Aristotle University, 54124 Thessaloniki, Greece; operdent@gmail.com; 5 Center for Adolescent Medicine and UNESCO Chair on Adolescent Health Care, First Department of Pediatrics, School of Medicine, National and Kapodistrian University of Athens, Aghia Sophia Children’s Hospital, 11527 Athens, Greece; bacopouf@hotmail.com; 6 Department of Pediatric Cardiology, Onasseio Cardiac Surgery Center, 17674 Athens, Greece; ahoursal@yahoo.gr; 7 Department of Biochemistry and Biotechnology, University of Thessaly, 41500 Larissa, Greece; dkouret@uth.gr; 8 Division of Dental Tissues’ Pathology and Therapeutics (Basic Dental Sciences, Endodontology and Operative Dentistry), School of Dentistry, Aristotle University Thessaloniki, 54124 Thessaloniki, Greece; nikolchem@dent.auth.gr

**Keywords:** chemical hazards, safe use, labelling

## Abstract

Over the last decades, human activities prompted the high production and widespread use of household chemical products, leading to daily exposure of humans to several chemicals. The objective of this study was to investigate the frequency of chemicals’ use by children and parents in Greece and estimate the level of risk awareness and understanding among them. A total of 575 parents and children were asked to answer an anonymous, closed-ended, validated, and self-administered questionnaire. One-third of the children and almost half of the parents participating in the study believed that commonly used chemical products do not pose any risk to human health or to the environment, despite the product labelling. The majority of both children (61.8%) and parents (70.6%) were informed about product safety via the product labelling. Around 20% in both groups could not differentiate between systemic toxicity and acute lethal effects depicted by pictograms on the label and milder hazards, such as skin irritation. Moreover, the information on hazard and precautionary statements appearing on the label was very poorly perceived. Therefore, as both children and parents seem not to clearly identify the hazards and risks arising from the use of everyday chemical products, targeted awareness policies should be implemented to support the safe use of household products.

## 1. Introduction

The increasingly high rates of manufacturing of commercial chemicals and the widespread use of industrial products are leading to an increase in the daily exposure of humans to chemicals. Therefore, the public becomes more vulnerable to the inherent human health hazards of various chemicals, along with any interaction and synergistic effects from combined exposure [1,2]. Children and infants are of especially great concern and are regarded as sensitive target groups for many reasons [3]. As they usually exhibit attitudes like hand-to-mouth movements, mouthing or chewing items, and playing on the ground, increased exposure via systemic routes and dermally to several chemicals not easily recognized (“silent chemicals”) can be expected [4]. In addition, a series of various actors of their developing physiology (e.g., how they absorb, distribute, and metabolize chemicals, the level of oxygen consumption, water intake, eating patterns) [3,5,6] may affect children’s resistance to chemical hazards. Moreover, their specific response against chemical risks is likely associated with the rapid growth and development of the human body during childhood, which has been taken into account by several researchers [7,8,9]. A higher period of time is also available for young children to develop chronic diseases caused by toxic chemicals [10], while their immune system is quite immature with regard to withstanding such invasions [11]. 

A huge variety of everyday household items could be considered potential sources of exposures to numerous chemicals and mixtures thereof, through potential carriers like air, dust, and food [12,13,14,15]. Endocrine-disrupting chemicals (EDCs) are well-known as chemical compounds which can cause endocrine system disorders leading to adverse health effects in human organisms [13]. Organophosphate and pyrethroid insecticides, bisphenol A (BPA), as well as phthalates and polybrominated diphenyl ether flame retardants are some of the most common representatives of EDCs [16]. In particular, BPA is widely used in the production of thermal print papers, food, and beverage containers [17,18,19], and as a flame retardant in furniture and electronic equipment [20,21]. Phthalates are often present as plasticizers in PVC and vinyl flooring [22], and are detected in cosmetics and personal care products (soaps, shampoos, lotions) [23], medical supplies, food packaging, toys, and other plastics [24]. Both EDC and polycyclic aromatic hydrocarbon (PAH) air pollutants have been blamed for the occurrence of metabolic diseases, like childhood obesity and type 2 diabetes [25,26,27]. PAHs derived from tobacco smoke have been associated with increased asthma risk [10,28,29]. Household cleaning products (such as bathroom, floor, glass, kitchen, tiles and leather cleaner chemicals, non-chlorinated and chlorinated bleaches, sanitizers, air fresheners, insecticides) are sometimes related to rhinitis symptoms in children [30].Household batteries containing heavy metals have been found to contain mercury or cadmium concentrations exceeding the limit values of the EU Batteries Directive, 2006/66/EC [31]. Mercury, nickel, lead, and cadmium have also been identified in jewelry and toys, increasing the risk of adverse neurodevelopmental impacts [32]. In particular, cadmium exposure may be followed by hearing loss risk and DNA methylations in preschool children [33]. Likewise, the unsafe storage of household medicines leading to unintentional child poisoning represents a significant public health problem [34].

Therefore, health effects due to exposure to household products in children inevitably attracts the interest of the scientific community. Nevertheless, exposure itself, which is primarily linked to the use of chemical products, has not been extensively studied. At the same time, chemical products bear labelling according to European Regulations [35,36,37] in order to communicate their recognized chemical hazards to consumers and provide information on the safe use of the products. To what extent these labelling requirements manage to convey the message to users remains a matter of investigation. The present study aims at elucidating the use of chemical products in Greece regarding the kind of chemicals used by children and their frequency of use and exploring, for the first time to the authors’ knowledge, the extent of chemical hazard awareness by both children and their parents, and the impact of chemicals on children in Greece. There is scarce evidence on children’s exposure to chemicals in Greece. Developmental exposure to polyfluoroalkyl substances (PFAS) can contribute to pediatric liver injury [38]. The cumulative six-month incidence rate of childhood burn disease, chemical burns included, is 5% [39].Lead and mercury exposure remain a threat to optimal health for children [40,41]. References also exist that correlate chemical exposure with obesity in Greece [27]. According to the annual reports published by the General Chemical Laboratory of Greece [42], the conformity of commercial products and child-care products with regards to content and the migration of chemicals is around 90%. Nevertheless, exposure to multi-chemicals could raise concern for human health due to their synergistic effects [1].The significance of the current work relies on the potential contribution of the obtained findings not only to the improvement of children’s quality of life, but also to an increase in their average life expectancy.

## 2. Materials and Methods

A total of 575 individuals (235 parents and 340 children) from Athens, Thessaloniki, Larissa, and Korinthos, Greece, were asked to answer two (one for parents and one for children) anonymous, closed-ended, validated, and self-administered questionnaires with 23 and 25 close-ended questions, respectively, from May 2017 to December 2018. The questionnaire was distributed at the following centers collaborating in this study: the Centre for Adolescent Medicine and UNESCO Chair on Adolescent Health Care, National and Kapodistrian University; Aghia Sophia Children’s Hospital, in Athens; the School of Medicine and School of Dentistry of the Faculty of Health Sciences in the Aristotle University in Thessaloniki; the Department of Biochemistry and Biotechnology, University of Thessalyin Larissa; and a private pediatric clinic in Korinthos.

The questionnaire was left at the reception desks of the above-mentioned centers, accompanied by an explanatory opening page, where it was stated that the results of the survey were to be published. The return of the completed questionnaire was considered the written consent of the study population. The 575 individuals that returned the questionnaire correspond to an estimated return rate of 23.8% for children, in the range of the typical self-completed surveys, and around 12.9% for parents.

The questionnaires were developed at the University of Thessaly, Department of Biochemistry and Biotechnology (Larissa, Greece) in the framework of the MSc course on toxicology and were structured in two sections. The first section addressed demographic information (seven questions for both questionnaires) and the second investigated the risk/hazard communication of chemicals (23 questions for parents and 25 for children).The questions mainly focused on the use of chemical products (frequency, classes of chemicals, articles, etc.), hazard and risk of the chemicals (personal understanding/awareness of the hazard and risk for the human health and the environment), hazard and risk communication (labelling and packaging), information and education sources, prevention/accident treatment, and therapeutic approaches. Regarding the selection criteria, children 11 years old and above with no diagnosis on any kind of intellectual disorder or learning disability and their parents were enrolled in the study. Understanding of general information, symbols, the internet, and medical management issues increases significantly by the age of 12 for school-age children [43,44,45].

Once the questionnaire was constructed, a multidisciplinary group of professionals that were not participating in the present research group was asked to review the document and provide input. This expert group consisted of a toxicologist, two regulatory officers, a representative from the industry and a psychiatrist. The group provided input on the general content and face validity of the questionnaire (Content Validity Ratio-CRV =0.996, *p* < 0.05) [46], which was proven to be complete and adequate for distribution.

The 103rd General Assembly of Specific Interest (9 March 2016) of the Department of Biochemistry and Biotechnology, University of Thessaly, provided approval for the conduct of the study and distribution of the questionnaires [47].

Statistical analysis was performed using SPSS 22.0 software (IBM Corp., Armonk, NY, USA). Descriptive data were calculated as frequencies and percentages. Chi-square (*χ*^2^) tests were computed to reveal meaningful associations between chemical use and the categorical study variables (e.g., sex, level of education, geographical distribution), and Pearson’s correlation was performed for continuous variables (i.e., age). *p* < 0.05 was considered to indicate a statistically significant difference.

Study limitations: This study is not a biomonitoring study but is based on self-reporting. Therefore, no direct evidence of exposure to chemicals is provided, but rather an indication for possible exposure via the use of chemical preparations and articles. The study participants were not personally interviewed. Therefore, as the questionnaire was distributed to families, biased answers could not be excluded. Exclusion criteria of the study population including co-founding factors, such as parents’ profession, have not been used. The number of participants corresponded to a pilot study and the findings should be considered as such. In addition, the study population comes from a European Union (EU) country, where specific legislation (regulations) regarding chemical product use exist. European regulations on chemical products are in agreement with the Globally Harmonized System of Classification and Labelling of Chemicals (GHS) adopted by the United Nations (UN) in 2002. During the World Summit on Sustainable Development (Johannesburg, South Africa, 2002), countries were encouraged to implement the GHS as soon as possible with a view to having the system fully operational by 2008. Therefore, the results of this pilot study could represent a guidance tool for studying children and parents’ awareness regarding potential hazards derived from the use of chemical products world-wide. Nevertheless, an EU member-state-specific, or in general a nation-specific awareness policy, enforcement measures, and cultural habits could have a negative impact on the representativeness of the study results within the EU and world-wide.

## 3. Results

Detailed demographic data of the study population are presented in Table 1.

**Figure 1:**
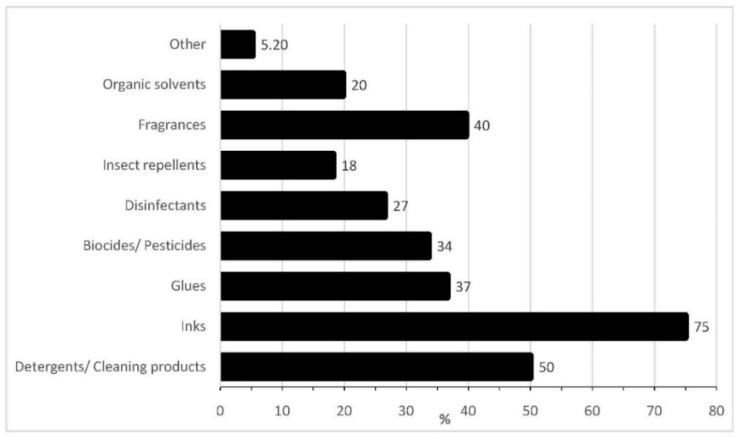
Popular chemicals used by children.

**Figure 2:**
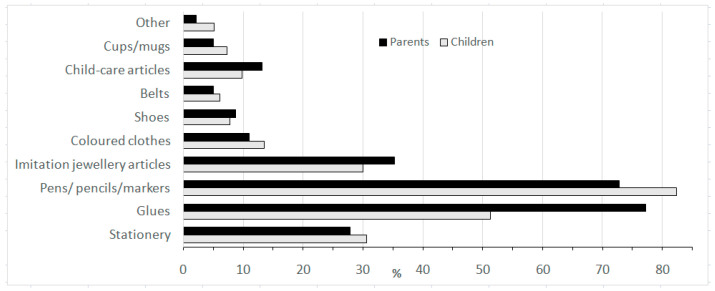
Exposure to chemicals via the use of articles as perceived by users (%).

With regards to the groups’ perception of their exposure to chemicals and from which sources, the responses showed that for many of the everyday products both groups are not aware of the existing chemical exposure and more specifically of the identity of the hazards. Regarding the use of chemical products, one-third of the children responded that they either do not use (10.4%) or that they are not aware if they even use chemicals (20.2%). On the contrary only 10.4% of the parents were not certain regarding the use of chemical products. However, when the questions related to more specific commercial products, such as adhesives, pesticides, detergents, petroleum products, etc., all responders were aware of the chemical nature of the respective product. Among children, stationery items, like pens, glues, inks, and detergents, were more popular; around 70% of the responders use them either rarely (9.80%), occasionally (35.6%), or daily (44.3%) (Figure 1 and Figure 2).

In Table 2, detrimental effects on human health that are anticipated by the users of chemicals, as reported by children and parents, are presented, but the users failed to connect specific chemicals/commercial products used with specific hazards to which they were exposed. The parents’ age and level of education are considered to have an important impact on their ability to comprehend and anticipate chemical risks (*p* < 0.001). The lower the parental age, the more the willingness to learn and draw information, while a high education level favors such behaviors to a high extent.

The use of chemical products by girls was found to be statistically more frequent and systematic compared to that of boys (*p* = 0.012). Furthermore, the former rather do not prefer to use personal protective equipment (e.g., gloves) against possible chemical threats, like dermal irritation or sensitization (dermatitis) (*p* = 0.023).

**Figure 3:**
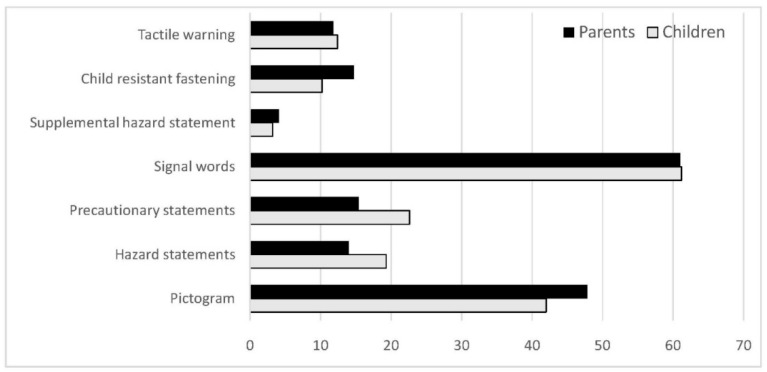
Perception (%) by the users of hazard communication on the packaging/label.

Labelling was mentioned as the main information source of communication of chemical hazards for both children (61.8%) and parents (70.6%), even though the users could not distinguish or fully understand the data on the label. One-third of the children questioned and almost half of the parents (47%) believe that the commercial chemical products they use do not pose a risk to human health or to the environment. From the items on the label, the most easily perceived is the signal word (i.e., “Danger” or “Warning”) and the pictograms (Figure 3). However, when the responders were asked to choose the pictogram depicting the most serious impact on human health (comparison between pictograms GHS05, GHS06, GHS07, GHS08), less than 5% of children (3.7%) and parents (3.2%) could actually understand the systemic effects communicated by GHS08 (chronic exposure, carcinogenicity, mutagenicity, reproductive toxicity, respiratory sensitization) compared to the acute lethal effects of GHS06 (skull and bones). All the above-described effects, along with dermal and eye corrosion (GHS05), and milder effects, such as irritation and sensitization (GHS07), were regarded as equally alarming with values of around 20% in both groups and no statistical impact from the level of education or age in the parent group. Similarly, when the groups were asked about the meaning of the phrase “poses a risk to human health,” the two groups showed statistically similar understanding; 69.5% of the children and 60.7% of the parents did not differentiate between lethal, carcinogen, toxic, and organ-specific toxicants, as far as the gravity of effects was concerned. Moreover, the warning for chemical hazards considered to be responsible for allergies, for example, was often undetectable or underestimated.

The communication channel which the two groups use to obtain information concerning hazards is the label of the product. Other channels less often used are the material safety data sheets (MSDS), the internet, and friends/family. Only 22% of the children participating in this study were informed about chemical risks associated with household products via educational programs in their school. The children themselves stated that drawing specific information on chemical risk through websites supported by experts or discussing risk communication with their classmates may increase the level of their relative awareness. It is important to mention that only 8.4% of the kids and 0.7% of the parents use the national competent authority (i.e., the General Chemical State Laboratory of Greece) as a source of information. However, when the groups were asked whether they would feel positively about consulting the national competent authority in the case of an emergency, the percentage was significantly higher (>85% for both target groups). Parents participating in the current research also stated that they would equally consult the National Organization for Medicines, the General Chemical State Laboratory of Greece, and the Ministry of Rural Development and Food about instructions for the use of chemical products. However, in case of an accident, children hesitate to address the Poison Centre in Aglaia Kyriakou General Hospital and they prefer to ask their parents for help. In contrast, parents show confidence in the medical community when facing such problems.

Finally, there have been recent labelling changes due to the new CLP Regulation (EC) No 1272/2008, which from 2008 to 2017 gradually replaced the directives Dangerous Substances Directive (67/548/EEC) and the Dangerous Products Directive (1999/45/EC), as far as classification and labelling is concerned. The study revealed that only 30.4% of the children and 20.3% of the parents have indeed noticed the labelling changes.

## 4. Discussion

Serious efforts have been made over the years by different national and international authorities and organizations in order to protect public health from chemical hazards. The Globally Harmonized System of Classification and Labelling of Chemicals (GHS) managed by the United Nations was set up to harmonize the perception of hazards globally. GHS utilizes harmonized criteria to classify substances and mixtures according to their health, environmental, and physical hazards and communicates hazards, including labelling requirements (hazard and precautionary statements, pictograms) and material safety data sheets (SDSs) [48]. Furthermore, the European Union (EU) incorporated in its regulatory system Regulation (EC) No 1907/2006 on Registration, Evaluation, Authorization and Restriction of Chemicals (REACH) [49] and Regulation (EC) No 1272/2008 on the classification, labelling and packaging of substances and mixtures (CLP) [50], aiming at the protection of human health and the environment from the hazards presented by chemicals and ensuring that chemical risks are comprehensively communicated to workers and consumers in the EU, respectively. The results of the present study on chemical risk awareness on behalf of children and their parents in Greece indicate that there is no clarity on important terms of legally requested labelling items depicting either the hazard of the preparation or its risk and how to be protected.

Although overall there is a good level of understanding among children and their parents on how to retrieve information about chemical products used in everyday life, as well as information on chemical substances expected to be present in chemical products and articles, it appears that the recognition of different hazards among the substances, the realization of the possible exposure to chemicals via products and articles, the use of personal protective equipment when advised by the manufacturers, and any possible risk management are still poorly perceived. Thus, regulators have to face the need to further develop the means of communication of hazards to the community and focus on the clarification of important terms of labelling for the safety of products. Furthermore, an effective communication framework consisting of scientists and policy makers should be developed addressing consumers’ needs, and especially vulnerable target groups, such as children. In this context, the call for proposals from the Commission for the European Partnership for the assessment of risks from chemicals (PARC) project within Horizon Europe funding, recently launched, seems to be aiming at the correct direction.

Children around thirteen years old were selected to be enrolled in the present study. In Greece, children’s monitored exposure to chemicals is not properly studied. Therefore, data on the use by children of chemical products and articles, which represent a constant source of exposure, may prove useful in organizing monitoring programs for specific chemicals. In addition, exploring how sensitive and informed the family environment is with regards to chemical hazards could provide extra insight on how protected children are from everyday chemicals. Our study showed that children use all kinds of chemical preparations available on the market, from cleaning products to insecticides, from before they are10 years old, even though their parents actually believe that the use of chemicals begins as their children become teenagers. Children and their parents appear to have very similar perceptions regarding exposure to chemicals via the use of articles, recognizing stationery and other items used at school as the main source of exposure. Therefore, exposure from other very commonly used everyday articles, such as mugs and clothing, is clearly underestimated and therefore protection from these sources is less effective. Market surveillance programs on such products and biomonitoring projects focusing on harmful chemicals contained in such products may contribute to a better protection of children’s health. The children of our study population appear a little more sensitive compared to their parents regarding serious expected detrimental effects on human health due to chemical use and subsequent exposure, such as cancer, whereas in general there is a common perception and worries within the family. The fact that the users failed to connect specific chemicals/commercial products used with specific hazards they are exposed to, in combination with the fact that a considerable percentage of users believe they are not using hazardous chemical products, could practically point to low awareness and educational input.

According to the World Health Organization (WHO), children are more vulnerable to the effects of exposure to chemicals. Globally, 54% of the burden of disease attributable to environmental exposures, expressed in disability-adjusted life-years (DALYs), is borne by children under the age of 15 years. Children cannot be regarded as little adults and are exposed to chemicals every day and throughout their lives [51]. The UN Environment Program (UNEP) has published a report that finds 25% of children’s toys contain harmful chemicals [52].Acute poisonings still cause mortality in the WHO European Region. The average mortality rate from unintentional poisonings in the region is 0.27 deaths per 100,000 population [51]. The unsafe use of chemicals resulting in accidental poisoning is fatal for more than 35,000 children annually, and even more do not result in death but cause permanent disabilities and diseases [53]. There is a growing recognition of the long-lasting effects of exposure to toxic environmental agents in early life, which can lead to diseases later in life; in the case of exposure to certain chemicals at critical life stages, impacts can even manifest themselves across generations. Acute and chronic, high and low-level exposures to chemicals in children’s environments may cause functional and organic damage during periods of special vulnerability.

The inability of children to recognize and interpret hazard warning signs plays a key role regarding the prevalence of their exposure to chemical attacks. It is crucial for the prevention of chemical accidents to identify the degree of chemical risk perception in children, as areas where children congregate are recognized as zones of high risk [54]. Home safety practices reducing child injuries (e.g., burns, poisoning, or drowning) can be effectively supported mainly through three practical approaches, including the removal of hazards and the use of safety equipment (environmental strategy), parental supervision, and face-to-face children’s education about safety rules and routine [55,56]. Moreover, parent education and training programs can ideally improve maternal psychosocial health, child behavioral problems, and parenting practices, tending to the further efficacy of parental interventions in preventing unintentional injuries in children [57,58].Undoubtedly, parents’ advisory interventions through a teaching process dealing with the realization that household products contain toxic chemicals can vitally contribute to the elimination of children’s exposure to chemical risks. Literature data related to the effectiveness of parental teaching about home safety showed that while the role of the parents is important in increasing children’s knowledge of human health hazards from chemicals, self-recognition of the hazard by the children and the following change in their attitude is equally important [59,60].

According to the recent opinion of the SCCS Committee of the EC [61], a decline in accidental ingestion and its adverse effects on children has been observed. This can be explained from different factors such as the development of safer formulation, packaging and storage, the increase in education of parents and caregivers about the risks and how to manage them in order to protect children, and updates in the legislation to prevent unsuitable containers (e.g., containers that are normally used to store food or drinks) being used to store harmful substances. It is generally accepted that child-resistant packaging is one of the best documented successes in preventing the unintentional poisoning of children [62]. However, they all agree that no matter how effective the aforementioned factors are, to remove the poison itself and replace it with other substances with a similar intended effect but with a lower toxicity still remains the most important element.

Identifying gaps in regulatory action on chemicals followed by a reduction in exposure are needed actions to mitigate the problem. Besides this, research into human health hazards across the life-span is needed to inform regulatory needs and appropriate interventions. Policies that promote integrated chemical management, comprehensive labelling, and marketing practices that incorporate child health considerations will enhance safe use. Towards this direction, informed healthcare providers play a key role in preventing and managing diseases [51].

## 5. Conclusions

In conclusion, the results of the present survey in Greece highlight several needs and interventions in order to ensure the safe use of chemicals by children and subsequent protection of their health. Namely, actual exposure data should be collected through biomonitoring programs and market surveillance projects on targeted chemicals at a national level. Moreover, educational programs in schools about the chemical risks of commercial products should be increased, and a thorough, informative campaign to parents should also be organized. Healthcare professionals should also be actively included in the loop of education and training by the relevant national authorities. Finally, regulators and policy makers in Europe should try and clarify the labeling requirements of commercial products, articles included, in order to more effectively communicate both hazards and risk-management options.

## Figures and Tables

**Table 1:** Demographics of the study population.

Demographics		% (*N*)
Demographics of Children		% (*N* = 340)
Sex	Male	42.5 (144)
	Female	57.5 (196)
Age		13.5 ± 3.2 (11–17)
Geographical distribution	Athens (capital)	30.9 (105)
	Thessaloniki (northern Greece)	25.0 (85)
	Larissa (central Greece)	8.8 (30)
	Korinthos (south Greece)	35.3(120)
No siblings		2.44 ± 1.05 (0–5)
Favorite lesson	Physicomathematics	48.2 (164)
	Humanitarian sciences	31.6 (107)
	Other	13.0 (44)
	None	7.3 (25)
Age of first use of a chemical product		8.58 ± 4.7
Demographics of Parents		% (*N* = 235)
Sex	Male	32.4 (75)
	Female	67.6 (160)
Age		40.5 ± 7.9 (31–52)
Geographical distribution	Athens (capital)	19.1 (45)
	Thessaloniki (northern Greece)	37.9 (89)
	Larissa (central Greece)	6.8 (16)
	Korinthos (south Greece)	36.2 (85)
No children		1.85 ± 0.84 (1–6)
Profession	Healthcare professional	9.6 (23)
	Education professional	8.8 (21)
	Other	58.1 (136)
	Unemployed	23.5 (55)
Education level	Primary	24.3 (57)
	Secondary	11.0 (26)
	Technical	27.2 (64)
	University	20.6 (48)
	Post-graduate	16.9 (40)
Age of first use of a chemical product by their children		15.4 ± 3.26

**Table 2:** Awareness of the study population regarding any possible effect of chemical product use on the human health.

Effects on Human Health Expected from Chemicals	Response	Children % (*N* = 340)	Parents % (*N* = 235)
Allergies	No	34.4 (117)	23.0 (54)
	Yes	65.6 (223)	77.0 (181)
Hepatotoxicity	No	88.3 (300)	91.3 (215)
	Yes	11.7 (40)	8.74 (20)
Reproduction	No	85.6 (291)	88.1 (207)
	Yes	14.4 (49)	11.9 (28)
Neurological effects	No	86.1 (293)	92.9 (218)
	Yes	13.9 (47)	7.13 (17)
Cancer	No	42.2 (143)	62.7 (147)
	Yes	57.8 (197)	37.3 (88)
Skin corrosion	No	61.1 (208)	38.9 (91)
	Yes	38.9 (132)	61.1 (144)
Respiratory/Lung effects	No	46.1 (157)	39.7 (93)
	Yes	53.9 (183)	60.3 (142)

## Data Availability

Data available on request due to restrictions e.g., privacy or ethical. The data presented in this study are available on request from the corresponding author. The data are not publicly available due to the fact that they are regarded as intellectual property of the Department of Biochemistry & Biotechnology, University of Thessaly. Upon request to the corresponding author the General Assembly of the Department of Biochemistry & Biotechnology, University of Thessaly will assess the request and provide permission to access the data.
